# Comparative Study on the Tensile and Compression Process of the Collapsible Ultra-Thin-Walled Composite Lens Rod

**DOI:** 10.3390/ma18173993

**Published:** 2025-08-26

**Authors:** Haitao Luo, Jia Fu, Yuxin Li, Chengzhi Ni, Haowen Tong, Peng Wang

**Affiliations:** 1 State Key Laboratory of Robotics and Intelligent Systems, Shenyang Institute of Automation, Chinese Academy of Sciences, Shenyang 110016, China; fujia@sia.cn (J.F.); liyuxin@sia.cn (Y.L.); nichengzhi@sia.cn (C.N.); thw1145141919810@163.com (H.T.); 2 University of Chinese Academy of Sciences, Beijing 100049, China; 3 School of Mechanical Engineering, Shenyang University of Technology, Shenyang 110870, China; 4 Department of Mechanical Engineering, Tsinghua University, Beijing 100084, China; wpqsxr1989@163.com

**Keywords:** collapsible lens rod, ultra-thin-walled composite materials, tensile and compression process, finite element analysis, spatial structure

## Abstract

In this paper, experimental data during the compression and stretching of ultra-thin-walled deployable composite lens rods are compared with the finite element analysis data to study the flattening and stretching. The advantages and disadvantages of the two compression methods in the process of compressing the lens rod are examined. The compression process of the lens rod can be considered a small strain process with nonlinear deformation. First, the composite lens rod model created by ABAQUS was established for a finite element simulation analysis of the compression and tension. Second, compression and tensile experiments were performed on the pod samples. Then, by comparing the measurement results and the corresponding finite element simulation results, we verified the accuracy of the finite element simulation method and experiment. In the compression flattening mode and the tensile flat mode, the maximum error between the simulated and experimental values is 8.3% and 8.7%. Finally, by comparing the maximum tension, displacement, and strain results of the two compression methods, the compression squash is smaller and more uniformly distributed than the tensile squash stress, and the squash method should be preferred in the design of the mechanism.

## 1. Introduction

With the continuous improvement of spacecraft performance and power consumption, the demand for lightweight and reliably deployable support structures is becoming increasingly prominent. The German Aerospace Center (DLR) [1] has developed a solar sail system based on ultra-light reflector wings and deployable sail trusses, and completed the verification in a zero-gravity environment through parabolic flight in February 2009. To meet the higher requirements for the stiffness and stability of the support structure of high-performance spacecraft, Liu [2] developed a new type of mechanism for the deployment and support of satellite solar wings using composite thin-walled lenticular tubes (CTLTs). It was successfully deployed to geosynchronous orbit in December 2019.

At present, many scholars have conducted research on the theoretical models and structural characteristics of composite material structures, and a number of innovative studies have promoted the development of this field. Meng [3] developed a three-dimensional (3D) finite element analysis (FEA) to investigate the effect of the fiber lay-up on the failure of laminated composites in bending. Liu [4] proposed a new analytical model to accurately predict the compressive behavior of composite helical structures (CHS). Sakovsky [5] proposed a dual matrix composite structure with a locally elastic composite hinge to make the composite have a smaller folding radius than conventional resin-based fiber-reinforced composites. Xu [6] manufactured five types of tubes by filament winding and experimentally investigated the impact resistance. Bai [7] used seven state-of-the-art genetic algorithms to optimize two conflicting objectives: maximizing the compressive stiffness while minimizing the weight. Sun [8] investigated finite element (FE) modeling of thin-walled columns to develop a comprehensive understanding of the crashworthiness of functionally graded thickness (FGT) under lateral impact. Tavassolimanesh [9] subjected composite tubes to transverse compression loading during flattening under three different filling conditions: empty tubes, polyethylene filled tubes, and polyurethane PTFE filled tubes. Sun [10] explored the bending collapse behavior and energy absorption capacity of net aluminum (Al), net (CFRP), and Al/CFRP hybrid tubes. Huang [11] investigated the bending collapse and impact resistance of a multi-unit aluminum/carbon-fiber-reinforced plastic (Al/CFRP) hybrid tube under quasi-static and dynamic loading. Liu [12] conducted quasi-static and dynamic axial extrusion tests on various carbon/epoxy resin composite tubes. Swolfs [13] explained the basic mechanisms of these hybrid effects and described the state-of-the-art models to predict these effects. Morioka [14] proposed a novel method to simulate the deformation of thick fibrous materials given a three-dimensional model with a constant thickness and a known fiber orientation. Guo [15] first proposed a differential temperature forming process to fabricate steel/CFRP structures. Lopatin [16] considered the free vibration of a cantilevered composite cylindrical shell. The analysis proved the excellent forming capability of the forming process for steel/CFRP materials. Supian [17] used a unique method to study the hybridization effect in metal/synthetic fiber composites that were used as energy absorbing tubes. Yadav [18] investigated the buckling behavior and imperfection sensitivity of thin steel cylindrical shells in pure bending with a focus on a specific range of slender lengths that are commonly found in energy structures. Yan [19] fabricated empty and polyurethane foam-filled natural linen fabric-reinforced epoxy resin composite tubes using a hand-gluing process. Leclerc [20] presented the autoclave fabrication process of ultrathin composite booms and experimentally studied the behavior of three test samples. Shin [21] studied the damage behavior of aluminum-composite hybrid beams using finite element analysis (FEA) under a three-point bending load. Jalalvand [22] proposed a new FE-based method to simulate all possible damage modes of glass/carbon UD hybrid laminates under tensile loading. Wang [23] maximized the energy absorption of aluminum/CFRP hybrid tubes of different configurations and validated them by comparing the damage modes and crashworthiness metrics with specialized experimental studies. Li [24] characterized the transverse extrusion behavior of circular aluminum, glass-fiber-reinforced plastic (GFRP) and CFRP tubes with different geometries (e.g., diameter and thickness). Chen [25] studied the fracture response of hat-shaped composite tubes under quasi-static three-point bending (TPB) and transverse compression (TC) conditions. Liu [26] proposed a Fourier series-based modeling approach to analyze the interactive buckling wrinkling behavior of inflatable beams. Zhang [27] discussed that the increasing use of fiber-reinforced plastics (FRPs) in industries such as aviation, marine, and automotive required monitoring the structural integrity of composite structures and materials. Eratbeni [28] designed and fabricated CFRP sandwich panels with diamond-shaped cores and experimentally and numerically investigated their modal properties by comparing them with conventional elliptical sandwich structures. A novel concept of mesh-reinforced membrane (MRM) was proposed by Tao [29]. The tensile collapse mechanism of the MRM was elucidated based on three distinct deformation stages.

Carbon-fiber-reinforced polymer (CFRP) laminates are extensively employed to manufacture deployable composite thin-walled lenticular tubes (CTLTs). The study of its behavior is the foundation for ensuring the reliability of the structure. Jia [30,31] presented an explicit dynamic procedure-based finite element framework to study the quasi-static bending collapse behavior of CTLTs. The boundary conditions and loads for pure bending tests were described in detail, and the appropriate analytical parameters were determined. Then, an automated finite element simulation scheme was proposed, which considered the difference between the high geometric nonlinearity and the linear and nonlinear buckling of composite materials and of CTLTs. Hu [32,33] investigated the results of experimental and numerical analyses of the compressive and tensile crush types of expandable thin-walled lens tubes. Then, the linear and nonlinear buckling analyses of numerical critical buckling loads of the composite thin-walled lens tube were compared with the corresponding experimental results. The numerical approach to simulate the buckling behavior of CTLT under axial compression was also validated. Chen [34] conducted experimental and numerical simulations of the flattening and wrapping of composite thin-walled lens tubes, where the effects of the geometric nonlinearities and contact interactions were also considered. Firth [35] developed a mechanism to control the unwinding of a rollable carbon fiber boom. A novel gear hub uses a high-torque spring to maintain tension on the webbing during extension and retraction. This tension eliminates blooming and obviates the need for circumferential rollers. Bai [36,37,38,39] investigated analytical solutions for the geometric and mechanical properties of ultra-thin-walled lens collapsible composite tubes (LCCTs) during fold deformation to understand the actual fold deformation phenomena and improve the structural behavior and practical applicability of LCCTs. They also developed an analytical model to predict the folding moment and ultimate curl radius of a lens DCB during folding based on the energy principle and strain principle. Using the nondominated ranking genetic algorithm III (NSGA-III), a multi-objective optimal design framework was proposed to optimize the lens DCB. Next, they proposed a simplified theoretical model to predict the position of the neutral segment of a flat stretchable composite boom in tensile deformation (DCB). Finally, Bai et al. investigated the thermal behavior of thin-walled deployable composite booms (DCBs) in a space environment using ground-based thermal vacuum tests and finite element analysis methods. Lee [40] explored the full design space of a double-walled composite CTM boom to assess the validity of the analytical model. Sickinger [41] addressed the engineering activities of a key project for an innovative solar sail propulsion technology: a lightweight deployable boom wrapped in a massive membrane structure in space. Zhang [42] studied the load-bearing and winding characteristics of thin-walled lens arms made of fiber-reinforced composites. Through experiments (free end loading and winding) and finite element analysis, the influences of parameters such as the number of layers, layer angles, fiber types and contents were investigated. From the above literature, it can be known that the current research on CTLT mainly focuses on the mechanical behavior of the structure after unfolding, while there is very little research on the mechanical behavior during the unfolding process. Especially, the research on the evolution relationship between stress and strain during the folding and unfolding process of CTLT is still in its infancy.

This study comparatively analyzed the flattening and tensile properties of CTLT during compression and tensile processes by combining experiments with finite element simulation. The advantages and disadvantages of two compression methods were discussed for the compression process of the mirror rod. A finite element model was established based on ABAQUS for simulation, and corresponding compression and tensile experiments were carried out [43]. The validity of the model and the experimental method was verified by comparing the experimental data with the simulation results. Ultimately, by comparing the maximum tensile force, displacement and strain results of the two compression methods, it was confirmed that the compression method generates less strain and shows a better effect in reducing the damage during the storage process of CTLT.

## 2. Theoretical Modeling

### 2.1. Geometric Model of Crush Deformation

**Figure 1:**
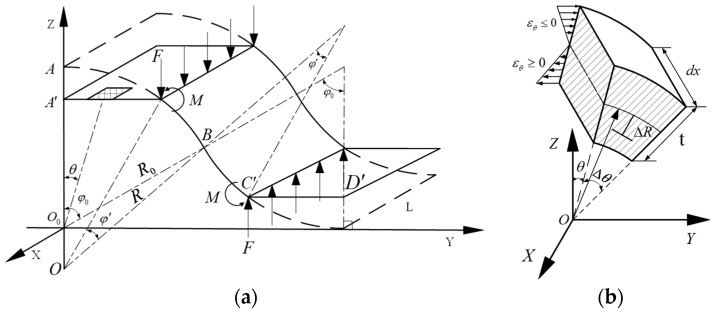
Compression deformation and geometric configuration of bean pod rod under a compression load. (**a**) Theoretical model; (**b**) microsome.

In Figure 1a, during the flattening deformation, the circular angle φ gradually decreases from the circular angle in the initial state to tend to 0, while the radius of curvature gradually tends to infinity from the radius of curvature at the initial state. Therefore, the displacement of point B with respect to the horizontal *y*-axis is [44]:

(1) $$Z_{B}=R(1-\cos\varphi )$$

Since the tangent arcs and have identical radii of curvature and circular center angles, the displacement from vertex A to the *y*-axis due to the deformation of the squash is:

(2) $$Z_{A}=2Z_{B}=2R(1-\cos\varphi )$$

Neglecting the wall thickness change of the pod rod during the folding deformation, the overall deformation can be described by the change in shape and radius of curvature of the neutral surface, which is not stretched. Thus, it is possible to obtain:

(3) $$R^{\prime }\varphi^{\prime }=R\varphi$$

According to the definition of elastic strain and Figure 1b, the in-plane strain in the direction tangential to θ of the pod rod during the flattening deformation can be expressed as:

(4) $$\varepsilon_{\theta }=\frac{\left(R+\Delta R\right)\varphi^{\prime }-\left(R+\Delta R\right)\varphi^{\prime }}{\left(R+\Delta R\right)\varphi }=\frac{\left(R+\Delta R\right)\varphi^{\prime }}{\left(R+\Delta R\right)\varphi }-1$$

where

$\varepsilon_{\theta }$: strain in the direction of the theta tangent during the flattening deformation of the pod rod;

Δr: change in radius of curvature of the neutral axis of the pod rod cross-section.

Substituting Equation (3) into Equation (4), we obtain:

(5) $$\varepsilon_{\theta }=\frac{\varphi^{\prime }}{\varphi }+\frac{R\left(\varphi^{\prime }-\varphi \right)}{\varphi_{0}\left(R+\Delta R\right)}-1$$

The strain in Equation (5) is a function with respect to the variables *φ* and ∆*r*. According to Equation (5), the in-plane strain in the direction tangential to θ at the fully compressed state under the compressive deformation (*φ* → 0, r → ∞) can be derived as

(6) $$\varepsilon_{\theta }^{*}=\left\{\begin{array}{c} \frac{-\Delta R}{R+\Delta R}\\ \frac{-\Delta R}{R-\Delta R}\\ 0 \end{array}\right\}\;\begin{array}{c} \overset{⌢}{A^{\prime }B^{\prime }}\\ \overset{⌢}{B^{\prime }C^{\prime }}\\ \bar{C^{\prime }D^{\prime }} \end{array}$$

The strain in Equation (6) is simply a function of the variable ∆r.

### 2.2. Analytical Solution for the Crush Deformation of the CFRP Material Beanpole

According to the basic definition of bending moment, the acting bending moment along the *x*-axis direction on the micro-body (Figure 1a) can be expressed as:

(7) $$M=-\int_{-\frac{t}{2}}^{\frac{t}{2}}\left(E_{\theta }\varepsilon_{\theta }\Delta R\right)d\Delta R$$

Substituting Equation (5) into Equation (7), we obtain:

(8) $$M=-\int_{-\frac{t}{2}}^{\frac{t}{2}}\left[E_{\theta }\frac{R\left(\varphi -\varphi^{\prime }\right)}{\varphi \left(R+\Delta R\right)}\Delta R\right]d\Delta R-\int_{-\frac{t}{2}}^{\frac{t}{2}}\left[E_{\theta }\left(\frac{\varphi^{\prime }}{\varphi }-1\right)\Delta R\right]d\Delta R$$

According to the symmetry of the laminate and integral nature of the parity function, we obtain:

(9) $$\int_{-\frac{t}{2}}^{\frac{t}{2}}\left[E_{\theta }\left(\frac{\varphi^{\prime }}{\varphi }-1\right)\Delta R\right]d\Delta R=0$$

To make the description of Equation (9) more concise, Equation (9) can be changed to

(10) $$M_{x}=\frac{R\left(\varphi^{\prime }-\varphi \right)}{\varphi }\int_{-\frac{t}{2}}^{\frac{t}{2}}\left(E_{\theta }\frac{\Delta R}{R+\Delta R}\right)d\Delta R$$

Figure 1a shows the forces that act on the pod rod halves of the CFRP material. According to the equilibrium condition of the forces in the *z*-axis direction for the entire halves, we can deduce that

(11) $$F=\frac{P}{2}$$

Similarly, according to the moment balance condition, we obtain:

(12) $$P=\frac{2M}{R\left(1-\cos\varphi^{\prime }\right)}$$

Using Equations (3), (10) and (13), we can solve for the shape parameters *φ* and r and obtain the vertex deformation of the collapsible composite pod rod using Equation (3). Thus, the load–displacement curve (P-δ) of the CFRP material pod rod subjected to compressive load P in the vertical direction can be established.

## 3. Numerical Investigation

### 3.1. Compression Flattening Process of the CTLT

The structure of CTLT can be simply summarized as two open cylindrical shells joined together. Due to its circumferential geometric closure, it has higher torsional stiffness and overall stability compared to a single open cylindrical shell. The lens-shaped cross-section enables it to have a more uniform load distribution and lower stress concentration. The tube can be manufactured by bonding the upper and lower plates with a hollow, thin-walled lenticular cross-section. For lenticular sections, the process of compression and unfolding can be described as follows. First, vertical pressure can be applied through a compression mechanism. Then, the contact between upper and lower parts of the CTLT cross-section gradually extends from one point to a line. In this process, the contact between upper and lower parts develops from two bonded edges to the central region. Although the CTLT undergoes a large deformation in the case of full contact between upper and lower parts, the deformation cross-section of the CTLT can still return to its initial shape because the material of the CTLT has only a small strain in this stage.

The lens rod is 100 mm long and 0.15 mm thick. In subsequent experiments, a compression plate with dimensions of 100 mm × 100 mm is defined as a rigid body for flattening a pod-like cross-section. For boundary conditions of the finite element model, the peak of the central arc should be constrained in the x-direction, while the two combined edges on both sides should be fixed in the y- and z-directions. Numerical simulation compression plates only move vertically. The CTLT has a base plate size of 100 mm × 100 mm. Due to the symmetry of its geometric dimensions and the loading state with respect to the horizontal x-z plane, its simplified finite element model was created using the ABAQUS 2022 software with symmetry boundary conditions. When the compression plate moves downward, the contact area between the compression plate and the section gradually increases from a line to a plane. The CTLT model is made with a three-layer laminate (45/−45/0) carbon-fiber-reinforced polymer with epoxy resin and a ±45-degree layer thickness of 0.06 mm, a 0-degree layer thickness of 0.03 mm, and a length of 100 mm. Laminate prepreg specimens are made of USN05400 and USN03000 carbon fiber and epoxy resin with volume fractions of 35–40% and 48–60%, respectively. Table 1 describes the material properties of each layer of CTLT. Each square element has a length of 1.0 mm and 14,100 grids. The entire flattening process of CTLT includes nonlinear deformation and small strain.

### 3.2. Tensile Flattening Process of CTLT

The cross-sectional shape of the CTLT is lenticular. The process of putting away and unfolding the CTLT can be described as follows: First, a tensile force is applied to both edges of the CTLT, which can elastically flatten the upper and lower bulges. When the tension is released, the squashed tube can return to its initial shape.

**Figure 2:**
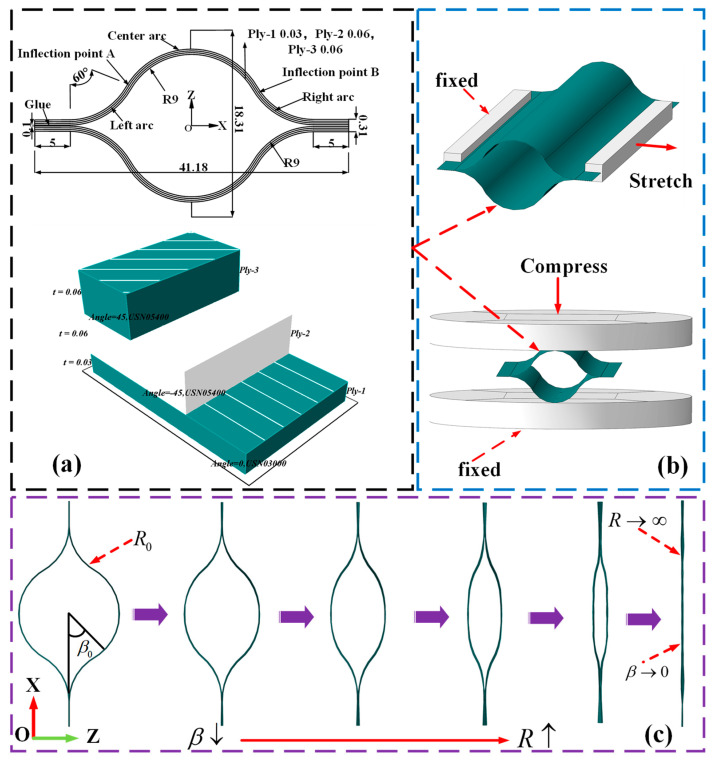
CTLT cross-section size, layup, and cross-section variation. (**a**) Cross-sectional dimensions and layups (mm), (**b**) stretch and compression models, (**c**) cross-section variation.

Figure 2a depicts a finite element model of the CTLT tensile flattening process. For the finite element model, the material properties of each layer can be referred to Table 1. The fixture is 80 mm long and 5 mm wide and simulated by a rigid body. The connection between two fixtures and two edges of the CTLT specimen is numerically simulated by beam connection. One clip is completely fixed, while the other clip can only move horizontally. During tensile flattening, the inner surfaces of the upper and lower side arcs first touch each other and subsequently gradually extend to complete flattening when the fixture moves horizontally.

## 4. Experiments

### 4.1. Compression and Flattening Experiment

**Figure 3:**
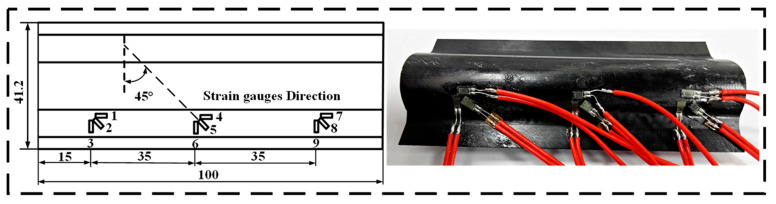
Tube sheet stretching and flattening device with a strain gauge.

In order to obtain the mechanical and geometrical nonlinear properties of the CTLT during flattening, it is necessary to perform flattening experiments on the composite lens tube. Strain gauges can be placed on the sample according to the layout in Figure 3. The compression tester is homemade [45]. The strain measurement system is a DHDAS dynamic signal acquisition and analysis system produced by DONGHUA Company in China, which is used to collect and analyze electrical signals. In this paper, it is used to collect the output signals of strain gauges. The strain gauges are arranged on the CTLT in the manner shown in Figure 3. The geometrical data of the CTLT specimen is shown in Figure 3. The CTLT specimen can be divided into three layers (45/−45/0) with the material parameters as shown in Table 1. The thickness of the ±45° layer is 0.06 mm, the thickness of the 0° layer is 0.02 mm, and the length of the CTLT is 100 mm. The cross-sectional variation of the CTLT specimen is shown in Figure 3, with the displacement of the tensile fixture and the compression plate, the R of the CTLT specimen gradually tends to infinity and gradually tends to 0.

**Figure 4:**
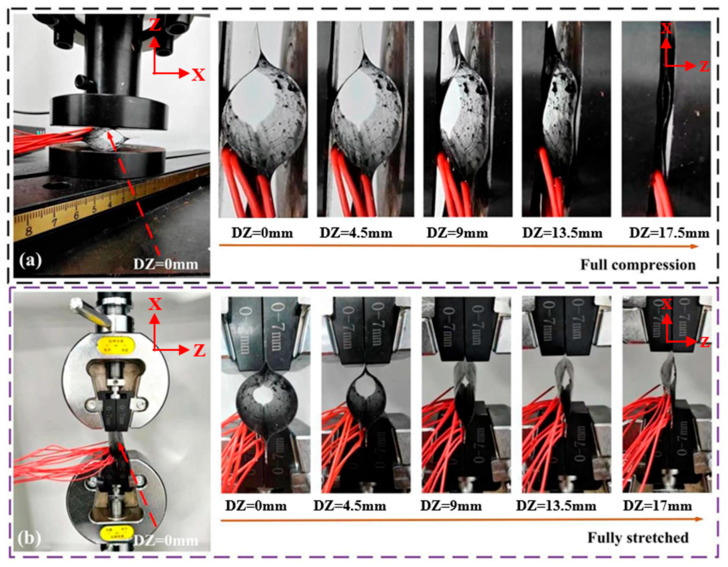
Localized depression in compression crush test. (**a**) Compression process, (**b**) stretching process.

The flattening experiments can be described as follows:

(1) As shown in Figure 3, due to the symmetry of the CTLT specimen, nine unidirectional strain gauges were pasted at the inflection points of one side of the arc.
(2) To keep the specimen in a stable flattened state, the specimen should be horizontally placed.
(3) The downward displacement of the compression plate is 0.5 mm/s. When the contact reaction force is 0.5 N, the test system starts to record the load and downward displacement. The total downward displacement is 17 mm. The entire flattening process is divided into 18 steps. Then, the experimental strain magnitude is obtained from various strain gauges. Figure 4a shows the compression and flattening process of the CTLT specimen.

### 4.2. Compression and Tensile Test

To obtain the mechanical properties of the CTLT specimens during the tensile flattening process, the tensile flattening process of the CTLT specimens should be measured. The CTLT segment should be stretched using a homemade tensile compressive test machine, and the experimental data should be collected using a dynamic signal acquisition and analysis system called DHDAS [46]. Figure 3 shows the tensile crush setup of the tube sheet with a strain gauge. The CTLT specimen consists of three laminated layers (0°/45°/−45°), where the ±45° layer is 0.06 mm thick, the 0° layer is 0.02 mm thick, and the section length of 100 mm. Table 1 shows the material properties of the CTLT specimen.

The strain gauges are pasted on the surface of the CTLT specimen, and Figure 3 shows their layout. Figure 4b depicts the tensile flattening process of the CTLT specimen, which is as follows:

(1) As shown in Figure 3, due to the symmetry of the CTLT specimen, nine unidirectional strain gauges were pasted at the inflection points of one side of the arc.
(2) To avoid relative sliding between fixture and section, the strain gauges can be tightly fixed by applying a transverse pressure to the screw fixture.
(3) Apply the displacement load at 0.5 mm/s and start recording the tensile displacement and the corresponding displacement load when the reaction force is 0.5 N. The tensile displacement and the corresponding displacement load are recorded when the reaction force is 0.5 N. The tensile displacement and the corresponding displacement load are recorded when the reaction force is 0.5 N. The first step was processed with a tensile displacement of 0.5 mm, then the tensile displacement was increased by 0.5 mm and loaded continuously for 12 s until the total tensile displacement reached 65 mm.

## 5. Results and Discussion

### 5.1. Finite Element Simulation Results and Analysis

**Figure 5:**
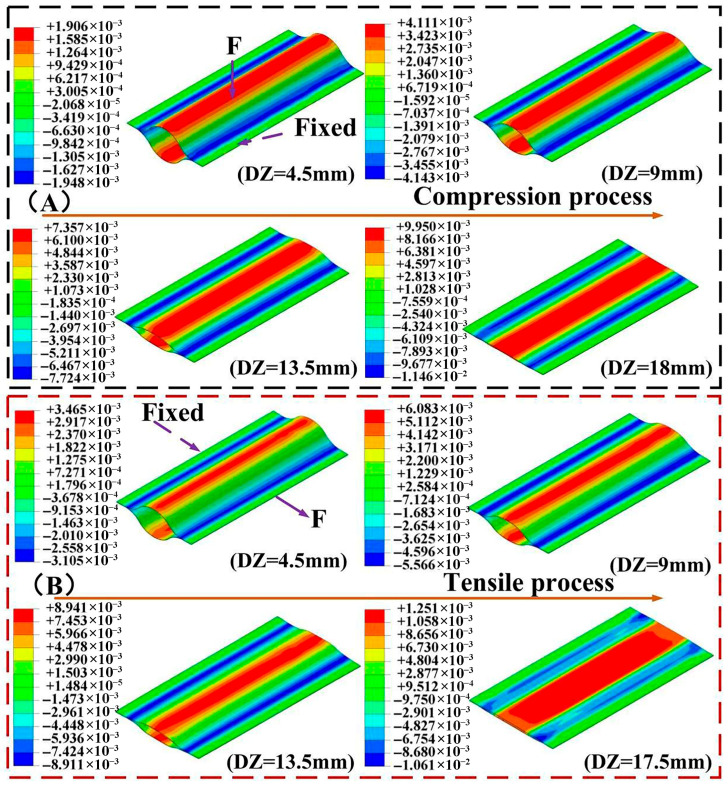
Contour plot of strain in x-direction during CTLT compression and tensile flattening. (**A**) Compression process, (**B**) stretching process.

Based on the abovementioned numerical simulation method, the flattening process of CTLT can be simulated. Thus, the behavior of the flattening process of CTLT can be obtained. Figure 5A shows the strain contour plot of the first layer of the CTLT flattening process in the Z-direction at 4.5 mm, 9 mm, 13.5 mm, and 18 mm displacements in the compressed version. According to Figure 5A, the tensile strain occurs on both side arcs, and the compressive strain occurs on the center arc. The strain on the central arc gradually increases with the increase in DZs. When the CTLT is completely flattened, maximum tensile strain occurs at both side arcs. The maximum strain of CTLT occurs when the CTLT is fully flattened, and the maximum tensile strain and maximum compressive strain are 0.01146 and 0.00995, respectively.

Figure 5B depicts the strain contour plot of the first layer of the CTLT tensile flattening process at tensile displacements of 4.5 mm, 9 mm, 13.5 mm, and 17.5 mm in the Z-direction (named “DZ”). In Figure 5B, the strain on both sides of the first cord layer is tensile strain, and there is a compressive strain on the center arc. The stress on the arc increases with the gradual increase in DZs and is symmetrically distributed on both sides of the arc in the length direction. CTLT specimens exhibit maximum strain when completely flattened. The maximum compressive strain occurs at DZ at 17.5 mm with a size of 0.01251.

**Figure 6:**
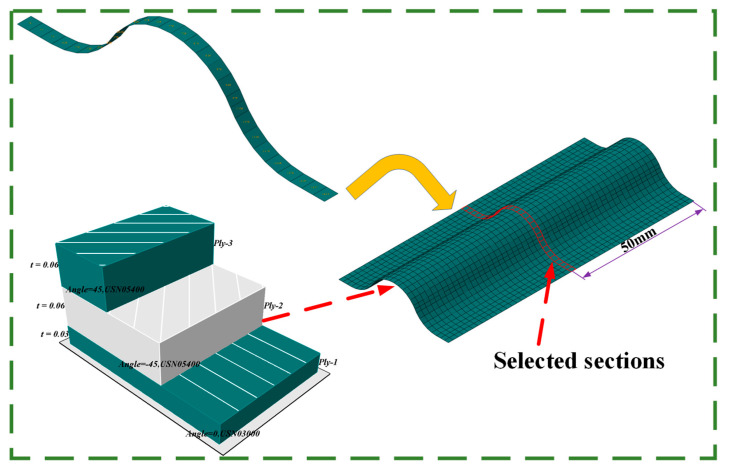
Selected cross-section in x-direction.

**Figure 7:**
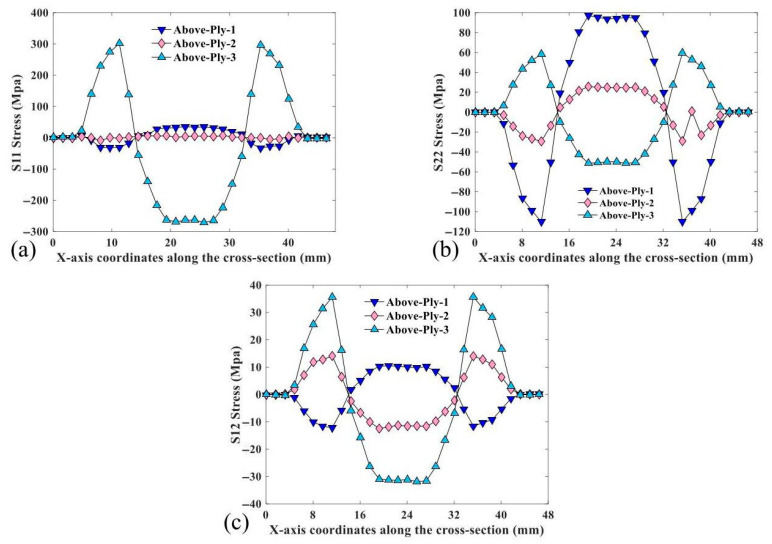
S11, S22, and S12 stress plots of selected cross-section of CTLTs in x-direction. (**a**) Compressed S11, (**b**) compressed S22, (**c**) compressed S12.

The CTLT cross-section 50 mm away from the CTLT side was selected to study the stress distribution. Figure 6 shows the selected cross-section of the CTLT. Figure 7 depicts S11, S22, and S12 of the selected cross-section of the CTLT in the x-direction. S11 is the principal stress in the fiber direction of each cord layer. S22 is the principal stress in the direction of the orthogonal fibers of each cord layer. S12 is the shear stress of each cord layer. As shown in Figure 7a, the tensile and compressive stresses of layer 1 occur at two side arcs and center arcs, respectively. The stress smoothly changes at both side and center arcs. The stress in the middle layer is basically 0 Mpa, and the maximum tensile and compressive stresses occur in the layer 3 at both sides of the arc and the center arc, with a size of 302 MPa and −271 MPa, respectively. According to Figure 7b, the maximum S22 is approximately 97 MPa. The tensile and compressive stresses of the second layer are present at the center arc and two side arcs, respectively. For S22, they are large in size on both side arcs and center arcs but very small at inflection points A and B. According to Figure 7c, the S12 shear stress on both edges of the CTLT is nearly zero. The maximum shear stress of 35 MPa occurs on the arc of the third layer, which is much smaller than the shear resistance of the polymer and shear strength of the CFRP laminate.

**Figure 8:**
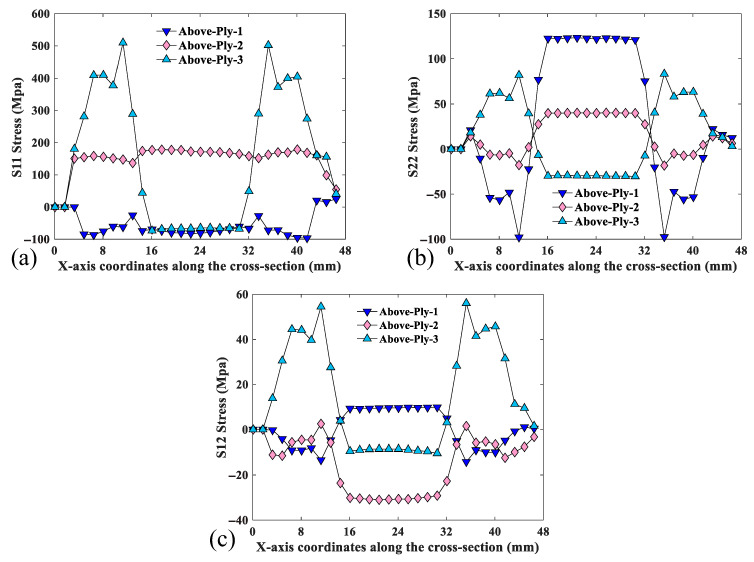
S11, S22, and S12 stress plots of selected cross-section of CTLTs in x-direction. (**a**) Tensile S11, (**b**) tensile S22, and (**c**) tensile S12.

The symmetric cross-section of the specimen segment is defined as the analytical target as shown in Figure 4. Figure 8 shows the S11, S22, and S12 stresses in the x-direction for the selected CTLT section. According to Figure 8a, S11 is the stress in the main direction of the tensile flattening process of the CTLT specimen, and the outer layer has the maximum tensile stress of 502 MPa at the position of the two side arcs, the middle layer exhibits tensile stress of about 150 MPa, and the inner layer exhibits compressive stress with the magnitude of −100 MPa; according to Figure 8b, it is the stress in the lengthwise direction of the CTLT specimen, and the maximum compressive and tensile stresses occur in the inner layer with the magnitude of −100 MPa; according to Figure 8c, S12 is the shear stress in the X–Y-direction of the CTLT specimen tensile flattening, and the maximum tensile shear stress occurs at the arcs on both sides of the external layer, with the size of 56 Mpa. Comparing Figure 7 with Figure 8, it can be clearly seen that the maximum stress caused by tensile mode is much larger than that caused by compressive mode in the process of flattening of the CTLT specimen, which indicates that the compression method is superior to drive the CTLT specimen flattening.

### 5.2. Comparative Analysis of Compression Experiment and Simulation Results

**Figure 9:**
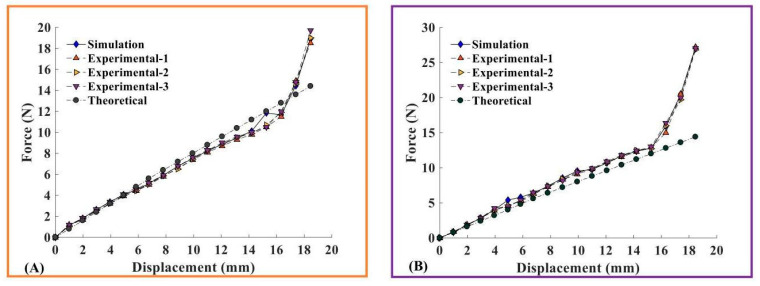
Compressive force curve increases with degree of flattening of the CTLT specimen. (**A**) Load and displacement relationships for compression flat patterns, (**B**) tensile flat mode load and displacement relationship.

The theoretical, simulation, and experimental comparison plots of the displacement of the specimen vertex along the Z-direction during compression and simulation of the CTLT specimen with the load it had been subjected to are shown in Figure 9. From Figure 9A, it can be seen that the displacement–load relationship of CTLT compression process can be divided into two parts: linear and nonlinear, in the linear part of the specimen had a uniform increase in the load with displacement, and when DZ = 16 mm, step into the nonlinear part, the small displacement of the specimen apex will lead to a sharp increase in the had a load, the maximum load of 19.7 N. The theoretical curve and the experimental value of the simulation curve coincide with the curve of the experimental value. From Figure 9B, it can be seen that the force required to stretch the specimen in the same Z-direction displacement is greater than that in the compression mode, and the maximum load is 27.8 N. The simulated value curve agrees well with the experimental value curve, and the theoretical value curve is slightly smaller than both. Overall, the theoretical model can be used to predict the compression process of CTLT, and the compression approach requires less load and is superior to the tension approach.

**Figure 10:**
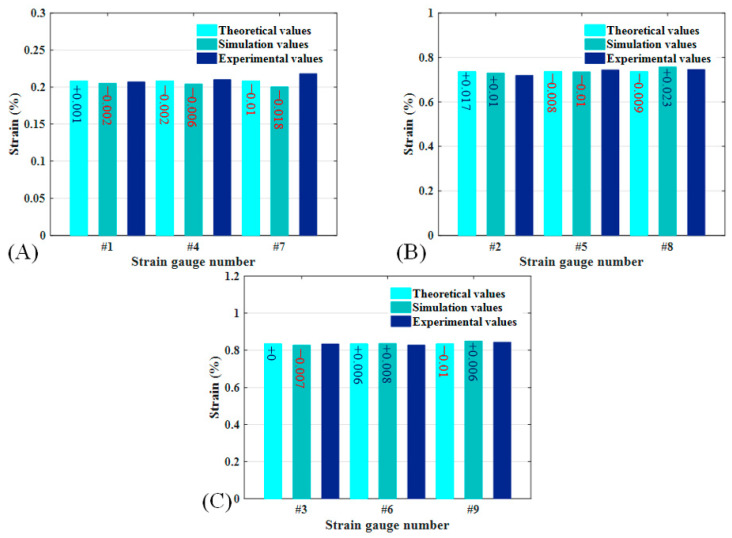
Strain values at each strain gauge position and direction for CTLT specimens fully flattened in compression. (**A**) Strain gauge #1, #4, and #7, (**B**) strain gauge #2, #5, and #8, (**C**) strain gauge #3, #6, and #9.

The strain values of the CTLT specimen during full compression are shown in the figure, including the theoretical model prediction, simulation analysis results, and three experimental values and their average values. The strain gauges were not damaged during the test, and the test data were accurate. The average of the three sets of experimental data is taken for comparison and analysis with the theoretical and simulation values. Figure 10A shows the axial strain of CTLT specimen, in the compression process, the theoretical, simulation, and experimental results of the strain gauge 1 and strain gauge 4 position compare well, but the experimental value of the strain gauge 7 position is too large, with the value of 0.00218; Figure 10B shows the fiber direction strain of the CTLT specimen, the theoretical, simulation, and experimental value of its each strain gauge position are more consistent, and the maximum fiber direction strain is the simulation value, with the value of 0.00768; Figure 10C shows the transverse strain of CTLT specimen, the theoretical, simulated and experimental values are in good agreement, and the maximum transverse strain occurs at the location of strain gauge No. 9 as 0.00849. Overall, the CTLT specimen has the maximum transverse strain, followed by fiber direction and the minimum longitudinal direction, and the theoretical model is the most accurate in predicting the value of transverse strain.

Table 2 demonstrates the strain values of CTLT specimens during the compression flattening mode, including the data cited in Figure 10 as well as the percentage of error between the theoretical model predictions and the experimental values, and the percentage of error between the simulated and analyzed values and the experimental average values. The maximum error between simulation and experiment is 8.3%, and the errors of other strain gauge attachment positions are smaller, and the strain value at this point did not fluctuate greatly during the three experiments, and the large error at this point is due to the residual glue on the surface of the strain gauge not being cleaned up completely, and in general the simulation model can simulate the compression experimental process of the CTLT specimen. The maximum error between the theoretical model and the experiment is 4.6%, due to the manufacturing error of the CTLT specimens used in the experiment, it can be considered that the constructed theoretical model can accurately predict the mechanical properties of the CTLT specimens in the compression process.

### 5.3. Comparative Analysis of Tensile Experiment and Simulation Results

**Figure 11:**
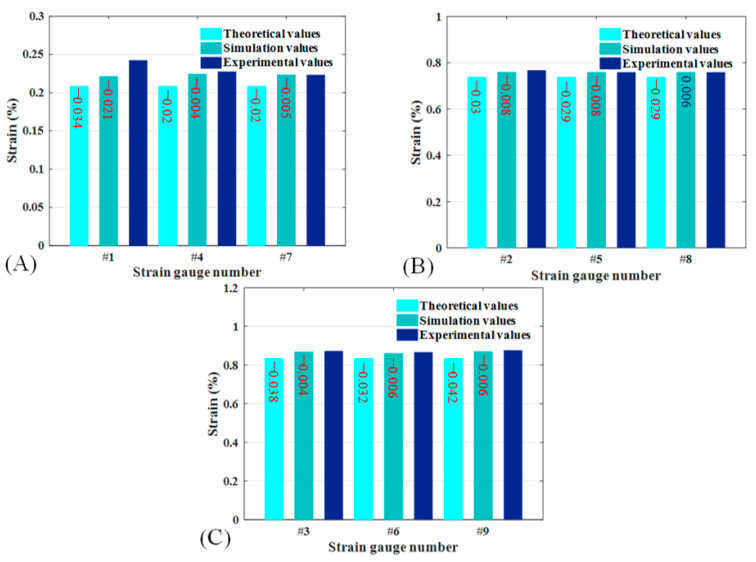
Strain values at each strain gauge position and direction for CTLT specimens fully flattened in tension. (**A**) Strain gauge #1, #4, and #7, (**B**) strain gauge #2, #5, and #8, (**C**) strain gauge #3, #6, and #9.

Table 3 demonstrates the strain values of CTLT specimens during the tensile compression method. The strain values of CTLT specimens in the complete tensile process are shown in Figure 11, including the theoretical model prediction, simulation analysis results, and three experimental values and their average values; the strain gauges were not damaged during the testing process, and the test data are accurate. Taking the average of the three experimental data and the theoretical and simulated values for comparison and analysis, Figure 11A shows the axial strain of CTLT samples. In the tensile process of each strain gauge position of the theoretical, simulation, and experimental results of the comparison of deviation, experimental and simulated values are greater than the theoretical value; the simulation and experimental values are closer to the experimental value of the strain gauge 1 position, which is the largest with a value of 0.00242. Figure 11B is the fiber direction strain of CTLT samples; the theoretical, simulated and experimental values of each strain gauge position are relatively consistent, and the maximum fiber direction strain is the simulated value of 0.00771. Figure 10C is the transverse strain of CTLT samples; the theoretical, simulated, and experimental values are relatively consistent, and the maximum transverse strain occurs in the position of strain gauge No. 9, which is 0.00875. Generally speaking, the transverse strain of CTLT samples is the largest, followed by the fiber direction; the longitudinal direction is the smallest, and the theoretical model has some deviation in the prediction of axial strain, but the prediction of transverse strain value is more accurate. By comparing Figure 10 and Figure 11, the tensile compression method produces larger strains than the compressive strains, which are more likely to cause damage.

This study verified the mechanical behavior of the ultra-thin-walled expandable composite mirror rod during the compression-tensile process through experiments and finite element simulation, confirming the superiority of the compression flattening method. It is worth noting that Zhang et al. [41] provided key design inspirations for the load-bearing and curling characteristics of similar thin-walled lens composite material booms: increasing the number of layers, raising the fiber content, and choosing low modulus fibers can significantly enhance the structural load-bearing capacity, but will weaken the curling ability. This literature further points out that although the orthogonal layup design can enhance the load-bearing effect, its influence weight is lower than that of the number of layers and the regulation of fiber parameters. The above findings collectively indicate that the design of thin-walled deployable structures in the aerospace field needs to strike a balance between load-bearing strength and deformation capacity (coiling/stretching). The compression flattening method proposed in this study provides a new idea for the performance optimization of such lightweight structures.

## 6. Conclusions

In this paper, a theoretical model of the compression and tensile flattening process of CTLTs is constructed. To verify the accuracy of the theoretical model, finite element simulation of the compression and tensile flattening process of CTLTs was carried out using the finite element software ABAQUS with the S4R shell unit considering nonlinear deformation, small strain, and contact interaction. A corresponding experimental study was carried out to investigate the flattening and tensile flattening process of the CTLT specimen and compare it with previous theoretical results based on the same geometry with the finite element. The numerical simulation results based on the same geometrical dimensions were compared with the theoretical results and the finite element model. The following conclusions are obtained.

(1) The force characteristics of each layer of the CTLT finite element model are analyzed, including the stress, strain, and pressure characteristics of each layer. The shear stress at the two side arcs is larger than other regions, the stress distribution is very uniform, and the shear stress at inflection point B is the smallest. At the beginning of the flattening process, the load grows slowly and the displacement grows rapidly, so the CTLT is easier to flatten. However, at the end stage of the flattening process, the load grows rapidly and the displacement changes slowly. In the numerical simulation of the flattening process, the shear stress on the arc is higher and the shear stress at inflection point B is the smallest.
(2) In the process of flattening and pulling, by the theoretical results and finite element analysis results, the linear relationship between strain and displacement is more obvious, and the growth rate of strain is very fast. According to the experimental collection of multiple sets of data, excluding the possibility of experimental errors, the strain experimental value does not have a relatively obvious nonlinear change, indicating that in the pod rod in the actual storage application, a larger load can be applied to completely flatten the pod rod to save storage space.
(3) In the experiments and simulations, the CTLT specimens are fabricated according to the geometry of their corresponding finite element models. The experimental and numerical simulations have good correlation in terms of load displacement and stress–strain variation with loading state, both of which reflect the validity of the theoretical model well. Both squashing processes are nonlinear deformation and small strain processes. The compression squash is smaller and more uniformly distributed than the tensile squash stress, and the squash method should be preferred in the design of the mechanism.

## Figures and Tables

**Table 1:** Composite parameters of the lens rod.

Property	USN05400	USN03000
Longitudinal modulus of elasticity $E_{11}$/GPa	116.62	141.98
Transverse modulus of elasticity $E_{22}$/GPa	8.9834	10.171
Cut the variable $G_{12}$/MPa	4377	3645
Cut the variable $G_{13}$/MPa	4377	3645
Cut the variable $G_{23}$/MPa	4000	3500
Poisson’s ratio $\mu_{12}$	0.29992	0.2923
Density ρ/(kg/$m^{3}$)	1600	1600

**Table 2:** Values of fully flattened strain of specimens in compression mode.

Strain Gauge Number (%)	#1	#2	#3	#4	#5	#6	#7	#8	#9
Theoretical value	0.208	0.736	0.833	0.208	0.736	0.833	0.208	0.736	0.833
Simulation value	0.205	0.729	0.826	0.204	0.734	0.835	0.200	0.768	0.849
Experimental value 1	0.206	0.724	0.838	0.218	0.744	0.827	0.224	0.754	0.847
Experimental value 2	0.198	0.721	0.840	0.212	0.751	0.824	0.217	0.756	0.835
Experimental value 3	0.218	0.712	0.821	0.199	0.737	0.830	0.213	0.724	0.846
Experimental mean value	0.207	0.719	0.833	0.210	0.744	0.827	0.218	0.745	0.843
Difference between simulation and experiment	0.001	0.017	0	−0.002	−0.008	0.006	−0.01	−0.009	−0.01
Difference between theory and experiment	−0.002	0.01	−0.007	−0.006	−0.01	0.008	−0.018	0.023	0.006

**Table 3:** Values of fully flattened strains of specimens in tensile mode.

Strain Gauge number	#1	#2	#3	#4	#5	#6	#7	#8	#9
Theoretical value	0.208	0.736	0.833	0.208	0.736	0.833	0.208	0.736	0.833
Simulation value	0.221	0.758	0.867	0.224	0.757	0.859	0.223	0.771	0.869
Experimental value 1	0.234	0.762	0.872	0.227	0.765	0.865	0.227	0.768	0.875
Experimental value 2	0.245	0.768	0.871	0.231	0.763	0.867	0.230	0.765	0.873
Experimental value 3	0.247	0.767	0.869	0.225	0.768	0.864	0.228	0.763	0.876
Experimental mean value	0.242	0.766	0.871	0.228	0.765	0.865	0.228	0.765	0.875
Difference between simulation and experiment	−0.021	−0.008	−0.004	−0.004	−0.008	−0.006	−0.005	0.006	−0.006
Difference between theory and experiment	−0.034	−0.03	−0.038	−0.02	−0.029	−0.032	−0.02	−0.029	−0.042

## Data Availability

The original contributions presented in this study are included in the article. Further inquiries can be directed to the corresponding author.

## Nomenclature

r	Radius of curvature of the neutral axis of the cross-section of the pod bar during the flattening process.
φ	Central angle of the neutral axis of the cross-section of the pod bar during the flattening process.
θ	Polar angle at any position of the cross-section of the bean pod rod in the polar coordinate system.
P	External compression load in the vertical direction of the center of the top arc of the pod rod.
$r_{0}$	Radius of curvature of the neutral axis of the cross-section of the pod rod in the initial state.
$\varphi_{0}$	Central angle of the neutral axis of the cross-section of the pod rod in the initial state.
$\varepsilon_{\theta }$	Strain in the direction of theta tangent during the flattening deformation of the pod rod.
Δr	Change in radius of curvature of the neutral axis of the pod rod cross-section.
$M_{x}$	*x*-direction of the restrained reaction moment.
$E_{\theta }$	Off-axis modulus of elasticity of the composite monolayer along the direction.
F	Restraint reaction force.
